# Supplementary material for: Inequalities in health system responsiveness among asylum seekers and refugees: A population-based, cross-sectional study in Germany
Source: PLOS Glob Public Health. 2022 Sep 28;2(9):e0000984. doi: 10.1371/journal.pgph.0000984 (PMC10021598; doi:10.1371/journal.pgph.0000984)
Supplement: S1 Fig — (DOCX) [file pgph.0000984.s001.docx]

**S1 Fig: STROBE chart representing sampling, data collection analysis process and number of individuals in reception centres and accommodation centres at each stage**

*
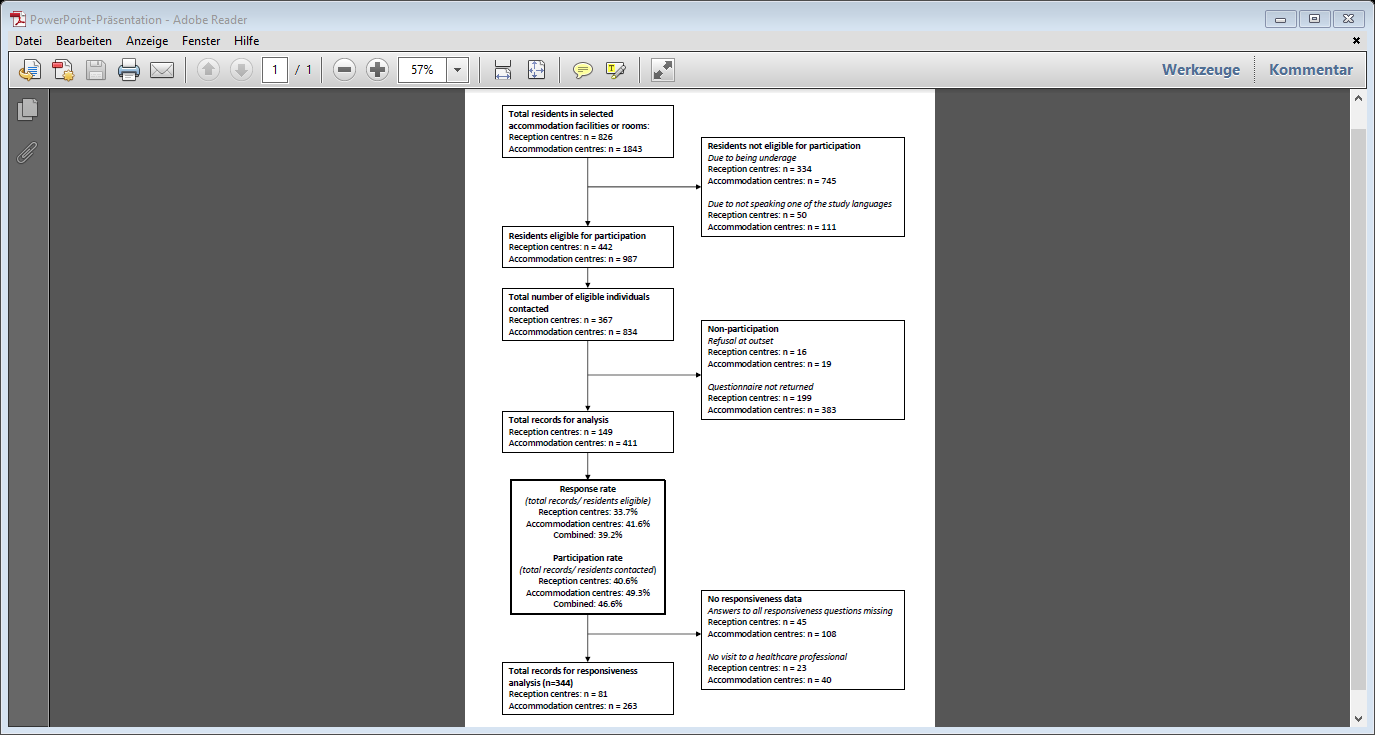
*
