# Supplementary material for: Inequalities in health system responsiveness among asylum seekers and refugees: A population-based, cross-sectional study in Germany
Source: PLOS Glob Public Health. 2022 Sep 28;2(9):e0000984. doi: 10.1371/journal.pgph.0000984 (PMC10021598; doi:10.1371/journal.pgph.0000984)
Supplement: S1 Table — (DOCX) [file pgph.0000984.s002.docx]

**S1 Table: Categorisation of educational score from survey items relating to school and continuing education**

|  | no further education | vocational training | university degree | don’t know |
| --- | --- | --- | --- | --- |
| no education | 1 | 1 | missing | 1 |
| still in school | 1 | missing | missing | 1 |
| mandatory schooling complete | 2 | 2 | 3 | missing |
| high school complete | 2 | 3 | 3 | missing |
| don’t know | missing | missing | missing | missing |
