# Supplementary material for: Inequalities in health system responsiveness among asylum seekers and refugees: A population-based, cross-sectional study in Germany
Source: PLOS Glob Public Health. 2022 Sep 28;2(9):e0000984. doi: 10.1371/journal.pgph.0000984 (PMC10021598; doi:10.1371/journal.pgph.0000984)
Supplement: S2 Table — (DOCX) [file pgph.0000984.s003.docx]

**S2 Table: Outcomes of logistic models of combined responsiveness with health factors and apriori confounders**

|  | *apriori confounders* | general health status  *& apriori confounders* | health limitation  *& apriori confounders* | longstanding illness  *& apriori confounders* | Health-related quality of life  *& apriori confounders* | PHQ2  *& apriori confounders* | GAD2  *& apriori confounders* |
| --- | --- | --- | --- | --- | --- | --- | --- |
| Average degrees of freedom | 30.146 | 30.300 | 29.830 | 30.022 | 29.566 | 29.933 | 29.796 |
| Model F-value | 0.660 | 1.179 | 0.604 | 1.534 | 1.789 | 2.463 | 4.205 |
| Model p-value (F-test) | 0.624 | 0.340 | 0.697 | 0.205 | 0.131 | 0.052 | 0.004 |
| Maximum FMI | 0.169 | 0.166 | 0.173 | 0.224 | 0.223 | 0.182 | 0.196 |
|  |  |  |  |  |  |  |  |
| Sex female (ref: sex male) | 0.71 (0.38,1.34) | 0.71 (0.37,1.38) | 0.73 (0.39,1.37) | 0.71 (0.36,1.40) | 0.66 (0.32,1.40) | 0.70 (0.37,1.32) | 0.79 (0.39,1.61) |
| Medium educational score (ref: lowest educational score) | 0.78 (0.31,1.96) | 0.70 (0.28,1.80) | 0.80 (0.32,2.01) | 0.78 (0.31,1.95) | 0.82 (0.32,2.18) | 1.00 (0.40,2.51) | 0.79 (0.30,2.14) |
| Highest educational score (ref: lowest educational score) | 0.82 (0.29,2.41) | 0.76 (0.26,2.26) | 0.80 (0.27,2.39) | 0.79 (0.27,2.29) | 0.78 (0.25,2.48) | 0.98 (0.33,3.00) | 0.71 (0.25,2.05) |
| Age at interview (linear) | 1.01 (0.98,1.05) | 1.02 (0.98,1.06) | 1.01 (0.98,1.05) | 1.02 (0.99,1.06) | 1.02 (0.99,1.06) | 1.02 (0.99,1.06) | 1.02 (0.99,1.06) |
| Bad/very bad general health (ref: moderate-v.good general health) |  | 0.43 (0.17,1.07) |  |  |  |  |  |
| Health-related limitation (ref: no health-related limitation) |  |  | 0.71 (0.29,1.71) |  |  |  |  |
| Longstanding illness (ref: no longst. illness) |  |  |  | 0.47 (0.23,0.98) * |  |  |  |
| Lowest HRQoL tertile (ref: highest HRQoL tertile) |  |  |  |  | 0.14 (0.04,0.51) ** |  |  |
| Medium HRQoL tertile (ref: highest HRQoL tertile) |  |  |  |  | 0.24 (0.07,0.82) * |  |  |
| Positive PHQ2 (ref: negative PHQ2) |  |  |  |  |  | 0.32 (0.16,0.68) ** |  |
| Positive GAD2 (ref: negative GAD2) |  |  |  |  |  |  | 0.22 (0.11,0.46) *** |

*FMI = fraction of missing information, HRQoL = health-related quality of life, PHQ2 = patient health questionnaire 2-item version, GAD2 = generalised anxiety disorder 2-item version*
