# Supplementary material for: Inequalities in health system responsiveness among asylum seekers and refugees: A population-based, cross-sectional study in Germany
Source: PLOS Glob Public Health. 2022 Sep 28;2(9):e0000984. doi: 10.1371/journal.pgph.0000984 (PMC10021598; doi:10.1371/journal.pgph.0000984)
Supplement: S3 Table — (DOCX) [file pgph.0000984.s004.docx]

**S3 Table: Outcomes of logistic models of combined responsiveness with socio-demographic factors and apriori confounders**

|  | *apriori confounders* | age binary *& apriori confounders* | subjective social status  *& apriori confounders* | West Asian nationality *& apriori confounders* | South Asian nationality *& apriori confounders* | West African nationality *& apriori confounders* | No. of social contacts *& apriori confounders* |
| --- | --- | --- | --- | --- | --- | --- | --- |
| Average degrees of freedom | 30.146 | 30.230 | 30.045 | 30.620 | 30.213 | 29.456 | 28.598 |
| Model F-value | 0.660 | 0.818 | 0.627 | 0.545 | 1.512 | 1.004 | 0.769 |
| Model p-value (F-test) | 0.624 | 0.523 | 0.708 | 0.741 | 0.212 | 0.430 | 0.600 |
| Maximum FMI | 0.169 | 0.172 | 0.175 | 0.166 | 0.160 | 0.226 | 0.276 |
|  |  |  |  |  |  |  |  |
| Sex female (ref: sex male) | 0.71 (0.38,1.34) | 0.68 (0.36,1.29) | 0.68 (0.36,1.30) | 0.71 (0.38,1.34) | 0.70 (0.37,1.32) | 0.73 (0.39,1.38) | 0.68 (0.35,1.33) |
| Medium educational score (ref: lowest educational score) | 0.78 (0.31,1.96) | 0.79 (0.32,1.98) | 0.75 (0.31,1.81) | 0.78 (0.32,1.92) | 0.67 (0.25,1.81) | 0.78 (0.31,1.95) | 0.74 (0.29,1.89) |
| Highest educational score (ref: lowest educational score) | 0.82 (0.29,2.41) | 0.82 (0.28,2.41) | 0.77 (0.28,2.12) | 0.82 (0.28,2.43) | 0.77 (0.25,2.38) | 0.79 (0.27,2.37) | 0.79 (0.26,2.38) |
| Age at interview (linear) | 1.01 (0.98,1.05) |  | 1.01 (0.98,1.05) | 1.01 (0.98,1.05) | 1.01 (0.98,1.05) | 1.02 (0.99,1.06) | 1.01 (0.98,1.05) |
| Age <31 years (ref: age 31+ years) |  | 0.64 (0.34,1.24) |  |  |  |  |  |
| Medium SSS in Germany (ref: low SSS in Germany) |  |  | 0.80 (0.29,2.23) |  |  |  |  |
| High SSS in Germany (ref: low SSS in Germany) |  |  | 0.59 (0.18,2.01) |  |  |  |  |
| West Asian nationality (ref: other nationalities) |  |  |  | 1.02 (0.46,2.25) |  |  |  |
| South Asian nationality (ref: other nationalities) |  |  |  |  | 0.41 (0.19,0.89) * |  |  |
| West African nationality (ref: other nationalities) |  |  |  |  |  | 1.89 (0.76,4.75) |  |
| no close personal contacts (ref: 3+ close personal contacts) |  |  |  |  |  |  | 0.45 (0.14,1.44) |
| 1-2 close personal contacts (ref: 3+ close personal contacts) |  |  |  |  |  |  | 0.63 (0.20,2.04) |

*FMI = fraction of missing information, SSS = subjective social status*
