# Supplementary material for: Inequalities in health system responsiveness among asylum seekers and refugees: A population-based, cross-sectional study in Germany
Source: PLOS Glob Public Health. 2022 Sep 28;2(9):e0000984. doi: 10.1371/journal.pgph.0000984 (PMC10021598; doi:10.1371/journal.pgph.0000984)
Supplement: S4 Table — (DOCX) [file pgph.0000984.s005.docx]

**S4 Table: Outcomes of logistic models of combined responsiveness with structural factors and apriori confounders**

|  | *apriori confounders* | residence status  *& apriori confounders* | health insurance card  *& apriori confounders* | accommodation *& apriori confounders* | urban-rural setting  *& apriori confounders* |
| --- | --- | --- | --- | --- | --- |
| Average degrees of freedom | 30.146 | 30.350 | 29.527 | 30.660 | 30.537 |
| Model F-value | 0.660 | 0.767 | 0.552 | 0.934 | 0.525 |
| Model p-value (F-test) | 0.624 | 0.601 | 0.735 | 0.472 | 0.756 |
| Maximum FMI | 0.169 | 0.168 | 0.203 | 0.168 | 0.172 |
|  |  |  |  |  |  |
| Sex female (ref: sex male) | 0.71 (0.38,1.34) | 0.69 (0.37,1.31) | 0.71 (0.38,1.36) | 0.71 (0.38,1.35) | 0.72 (0.39,1.36) |
| Medium educational score (ref: lowest educational score) | 0.78 (0.31,1.96) | 0.77 (0.32,1.90) | 0.79 (0.32,1.96) | 0.78 (0.31,1.96) | 0.77 (0.31,1.96) |
| Highest educational score (ref: lowest educational score) | 0.82 (0.29,2.41) | 0.83 (0.30,2.33) | 0.83 (0.28,2.45) | 0.83 (0.29,2.42) | 0.82 (0.28,2.40) |
| Age at interview (linear) | 1.01 (0.98,1.05) | 1.01 (0.98,1.06) | 1.01 (0.98,1.05) | 1.01 (0.98,1.05) | 1.01 (0.98,1.05) |
| Residence status: asylum granted  (ref: asylum seeker) |  | 0.51 (0.20,1.34) |  |  |  |
| Residence status: asylum rejected/  toleration (ref: asylum seeker) |  | 0.60 (0.24,1.52) |  |  |  |
| Health insurance card holder (ref: no health insurance card) |  |  | 0.87 (0.40,1.93) |  |  |
| reception centre resident (ref: accom. centre resident) |  |  |  | 0.59 (0.28,1.29) |  |
| urban setting (ref: rural setting) |  |  |  |  | 0.84 (0.42,1.70) |

*FMI = fraction of missing information*
