# Supplementary material for: Inequalities in health system responsiveness among asylum seekers and refugees: A population-based, cross-sectional study in Germany
Source: PLOS Glob Public Health. 2022 Sep 28;2(9):e0000984. doi: 10.1371/journal.pgph.0000984 (PMC10021598; doi:10.1371/journal.pgph.0000984)
Supplement: S1 Text — (DOCX) [file pgph.0000984.s006.docx]

**S1 Additional details of the methodological approach**

*Sampling weights*

The effect of the sampling weights is to correctly specify the variance of single point estimates to reflect the sampling design, which may be incorrect if single random sampling is assumed but the design is more complex. Despite a multi-stage sampling design, we follow West’s (2000) guidance for multi-stage surveys with small finite population corrections at the first level, weighting our dataset at the first level only (1). To combine the different sampling designs in reception centres and accommodation centres, we follow Kaminska & Lynn’s (2017) approach of treating these as separate clusters and separate strata in the weighting process (2). Data in both reception centres and accommodation centres are therefore clustered at the level of the facilities. While reception centres are treated as a single stratum, data in accommodation centres are stratified by (i) the number of refugees residing in each region and (ii) the size of the accommodation centre to reflect the sampling design.

*Single imputation for calibration*

To improve the model estimation of calibration variables, variables representing nationality, education, residence status, time since arrival and subjective social status (full list of variables in Table A) were also added to the predictive model. Imputation was carried out in two steps. First, the predictor matrix was set up and the imputation algorithm was chosen using predictive mean matching, polytome regression or logistic regression, depending on the variable (Table A). In a second step, the imputation matrix was set up with 30 iterations. The full data matrix was then used to calculate calibration weights. Data on gender, age and region of origin from the RESPOND sample, weighted to account for sampling design, was adjusted to the distribution of these variables among the population of asylum applicants using the iterative proportional fitting algorithm (raking-technique) (3). Calculation of calibration weights required 14 iterations until convergence. Imputation for missing values for the calibration as well as the calibration itself were carried out in R. The dataset was weighted in STATA using Taylor series linerarisation to estimate standard errors.

*Multiple imputation for analysis*

Variables were imputed based on linear, logistic, ordered logistic or multinomial regression models, depending on the variable scale (Table B). Data were augmented to avoid perfect prediction and required 300 iterations until convergence. A total of 30 datasets were imputed and analysed. Estimates were pooled across imputed dataset using Rubin’s rule (4).

*Assessment of multicollinearity*

Multicollinearity was assessed for the fully imputed dataset using the user-generated STATA command “collin” (5). Variance inflation factors were calculated for all independent variables used in the fully adjusted logistic regression models. Eigenvalues and Condition Index were calculated on the scaled raw score sums of squares and cross products (SSCP) matrix with an intercept.

*Table A: Variables used in single imputation model to calculate calibration weights*

| Variable | Type | Total missing | Model used for imputation |
| --- | --- | --- | --- |
| Age group | ordinal | 68 | Pmm |
| Sex | binary | 55 | Logreg |
| Nationality | categorical | 58 | Polyreg |
| Region of origin | categorical | 58 | Polyreg |
| Schooling | categorical | 82 | Polyreg |
| Study | categorical | 85 | Polyreg |
| Educational Score | ordinal | 166 | Pmm |
| Residence Status | categorical | 108 | Polyreg |
| Arrival in Germany | ordinal | 98 | Pmm |
| Subjective social status (Germany) | ordinal | 168 | Pmm |
| Subjective Social status (country of origin) | ordinal | 158 | Pmm |
| Date of entry | categorical | 98 | Polyreg |

*Table B: Variables used in multiple chained imputation model for statistical analysis*

| Variable | Type | Total missing | Model used for imputation |
| --- | --- | --- | --- |
| Age | continuous | 26 | Linear regression |
| Sex | binary | 17 | Logistic regression |
| Region of origin: western africa | binary | 22 | Logistic regression |
| Region of origin: southern asia | binary | 22 | Logistic regression |
| Region of origin: western asia | binary | 22 | Logistic regression |
| Educational Score | categorical | 82 | Multinomial logistic regression |
| Residence Status | categorical | 41 | Multinomial logistic regression |
| Subjective social status (Germany) | ordinal | 72 | Ordered logistic regression |
| Subjective social status (Germany) | ordinal | 67 | Ordered logistic regression |
| Quality of life | ordinal | 50 | Ordered logistic regression |
| No. of close social contacts | ordinal | 64 | Ordered logistic regression |
| Presence of health insurance card | binary | 41 | Logistic regression |
| General health status | binary | 24 | Logistic regression |
| Health limitation | binary | 40 | Logistic regression |
| Chronic illness | binary | 27 | Logistic regression |
| Positive PHQ2 | binary | 36 | Logistic regression |
| Positive GAD2 | binary | 36 | Logistic regression |
| Responsiveness: timeliness | ordinal | 0 | - |
| Responsiveness: respectful treatment | ordinal | 13 | Ordered logistic regression |
| Responsiveness:  communication | ordinal | 12 | Ordered logistic regression |
| Responsiveness:  autonomy | ordinal | 57 | Ordered logistic regression |
| Responsiveness: confidentiality | ordinal | 53 | Ordered logistic regression |
| Responsiveness: choice | ordinal | 101 | Ordered logistic regression |
| Responsiveness: cleanliness | ordinal | 17 | Ordered logistic regression |

*PHQ2 = patient health questionnaire 2-item version, GAD2 = generalised anxiety disorder 2-item version*

**References**

1. West B. Accounting for multi-stage sample designs in complex sample variance estimation. Suppl Mater Heeringa Etals Appl Surv Data Anal. 2010;

2. Kaminska O, Lynn P. Survey-based cross-country comparisons where countries vary in sample design: issues and solutions. J Off Stat. 2017;33(1):123–36.

3. Deming WE, Stephan FF. On a least squares adjustment of a sampled frequency table when the expected marginal totals are known. Ann Math Stat. 1940;11(4):427–44.

4. Rubin DB. Multiple imputation for nonresponse in surveys. Vol. 81. John Wiley & Sons; 2004.

5. Ender P. collin”: Stata command to compute collinearity diagnostics. 2010;
