# Supplementary material for: Inequalities in health system responsiveness among asylum seekers and refugees: A population-based, cross-sectional study in Germany
Source: PLOS Glob Public Health. 2022 Sep 28;2(9):e0000984. doi: 10.1371/journal.pgph.0000984 (PMC10021598; doi:10.1371/journal.pgph.0000984)
Supplement: Attachment — Submitted filename: response_to_reviewers.docx [file pgph.0000984.s007.docx]

Dear Editors,

Many thanks for your prompt and competent handling of our manuscript. We also thank the three reviewers for their positive and helpful reviews of our manuscript. We have made several amendments to our manuscript, as detailed below. In particular, we have added further details on the setting and the sampling approach to the methodology section and have condensed the discussion.

We hope that this revision meets the criteria for publication. If there are any further questions, please don’t hesitate to get in touch.

Kind regards,

Louise Biddle

| Please review your reference list to ensure that it is complete and correct. If you have cited papers that have been retracted, please include the rationale for doing so in the manuscript text, or remove these references and replace them with relevant current references. Any changes to the reference list should be mentioned in the rebuttal letter that accompanies your revised manuscript. If you need to cite a retracted article, indicate the article’s retracted status in the References list and also include a citation and full reference for the retraction notice. | We have reviewed the reference list and can confirm it is complete and correct. |
| --- | --- |
| Reviewer #1 |  |
| Important topic Need to clarify 15 vs 18mths for entitlements to care as the text says both. | Thank you for pointing this out. The different time periods are a result of a reform change in 2018. This has now been clarified in the methods section. |
| Good description of limitations and recognition the results may not reflect a deeper problem with access - I would add this to the abstract I am not able to comment on the data analysis for this paper as I am not sufficiently trained in statistics. It appears ok to me but I am hesitant to comment. | We have included a line in the abstract which indicates that further research is needed to understand the responsiveness of care for individuals who have not utilised health services and to disentangle expectation of care from responsiveness. |
| Reviewer #2 |  |
| This is an import and and well written article withe sound methodology. My comments are. | Thank you very much for your positive and helpful comments. |
| The discusion is well written but could be shortened | Thank you, we have identified several areas for condensing the discussion. However, based on the positive reviews of the discussion by the other reviewers we have avoided shortening the discussion section further. |
| The limitations of survey data in terms of its ability to reflect facts could be pointed out. This could be tied to those with long term chronic illness and also anxiety feeling they are not recieving responsive care. | We feel that we have adequately discussed the limitations of our data collection approach, including issues relating to non-response bias, imputation, combined responsiveness scores, the difficulties disentangling expectations of care from responsiveness and the need for further qualitative research to understand the reason for the observed inequalities. We have further pointed out that the WHO Responsiveness tool, despite being a survey approach, remains a valid and useful tool in providing an initial overview of assessing patient-rated quality of care and is able to point to areas for further inquiry and potential improvement -> no changes made. |
| Reviewer #3 |  |
| Summary Thank you for the opportunity I have to provide a review for this important article on inequalities in health system responsiveness among ASR in Germany. This article funded by BMBF focuses on assessing the quality of care among ASRs using the concept of responsiveness. The authors’ results and conclusions are quite informative and good for decision making by the ministry.  Cover page •The title clearly depicts the research that was carried out with focus on the outcome. •Each author’s contribution was clearly stated, and corresponding author’s contact is clear.  Abstract •The objective was to search if there were inequalities in health care among the population of studied and the summary on inequality findings was clearly written in the conclusion. •It is an interesting read for those who want to learn more about responsiveness in health systems.  Introduction •This aspect was well written and introduced the readers to the need to want to find out what the authors found out. | Thank you very much for your kind comments and detailed suggestions. |
| •However, the summary from reference 4 does not fully itemize the 5 goals of the Lancet commission, whose report was in the article. The authors can consider reviewing the sentence in relations to the article they quoted. | We have slightly adjusted this sentence to reflect the health issues discussed with reference to refugees and asylum seekers in the Lancet commission report under Section 3 “Burden of disease and migration” |
| •Readers were effectively introduced to the concept of responsiveness- this is very good  •The objectives are well stated at the end of the introduction | Thank you. |
| •Although I am of the opinion that the authors could have moved the last sentence in introduction to conclusion. | We agree and have moved this sentence to the conclusion. |
| Methods and Materials •I would advise the authors to provide the setting of the study to the readers of the journal. Not everyone knows the Germany’s third largest federal state, its population and the population of the ASRs. | We have added further details about the study setting at the beginning of the methods section. |
| •Sample size calculation was not stated, only data collection method was mentioned (“door to door approach”). Another researcher might not be able to repeat this method because of lack of guidance on how sampling size was determined. •The abstract stated that simple random sampling was done, but there is NO section on sampling technique in the methods and material section. | We describe our sampling approach in the second paragraph of the methods section. We have now added further details and also link to two publications which describe the sampling approach in detail. |
| •Data management was thorough and quite informative.  Results •Response rate was low, and it is good that the authors stated it. However, it has been compensated for in the statistical analysis. •The results of the 2 objectives of the study were clearly stated.  Discussion •The discussion is well written and provides the necessary reasoning behind the results of the study oEspecially when the researchers found a negative results in their study, they were able to provide probable reason for it  Conclusion •Provides answers to the two aims of the study •The recommendations made by the authors is appropriate for the study  References •The references are complete and well cited | Thank you. |
